# Use of prostheses in lower limb amputee patients due to peripheral arterial disease

**DOI:** 10.1590/S1679-45082014AO3132

**Published:** 2014

**Authors:** Therezinha Rosane Chamlian

**Affiliations:** 1 Escola Paulista de Medicina,Universidade Federal de São Paulo, São Paulo, SP, Brazil;; Lar Escola São Francisco, São Paulo, SP, Brazil.

**Keywords:** Lower extremity, Peripheral arterial disease, Amputation/rehabilitation, Amputees/rehabilitation, Peripheral arterial diseases/complications, Prostheses and implants, Mortality

## Abstract

**Objective:**

To evaluate the indication of prosthesis during rehabilitation and the maintenance of their use or abandonment rate after discharge, as well as mortality of lower limb amputees due to peripheral arterial disease.

**Methods:**

A retrospective and cross-sectional study carried out with lower limb amputee patients, at transfemoral and transtibial levels, due to vascular conditions. The sample was composed of 310 patients (205 men, 105 women, mean age 61.8 years), transfemoral (142) and transtibial (150) levels, unilateral or bilateral (18). A total of 217 were fitted with prosthesis and 93 did not. Nonparametric statistical tests with equality of two proportions, 95% confidence interval and p value <0,05 were used.

**Results:**

Out of 195 patients we contacted, 151 were fitted with prosthesis and 44 not. Of those that were fitted with prosthesis, 54 still use it, 80 abandoned and 17 died. In the group without prosthesis, 27 were on wheelchair and 17 died. Mortality is statistically higher among patients who were not fitted with prosthesis and 34 death occur, on average, 3.91 years after amputation. Survival time of patients who were not fitted with prosthesis was smaller than those were fitted.

**Conclusion:**

The use of prosthesis in lower limb amputees, due to vascular conditions, during rehabilitation is high. However, maintenance of prosthesis is not frequent after discharge. Early and high mortality is observed mainly among diabetic patients.

## INTRODUCTION

The number of lower limb amputee patients due to peripheral arterial disease is high in Japan, United States, Europe and Brazil, and the elderly population is the most affected.^(1-6)^

Despite advances in Medicine and the emphasis on disease prevention, amputations are still very prevalent in the world, and the projection is that by 2050 the prevalence rate will reach 3.6 million people in the United States alone.^(7)^

The mortality rates 1 month after amputation are high, ranging from 15 to 30%.^(8,9)^ After 1 year, the mortality rates are above 50%^(9)^ and, after 5 years, they may reach up to 74%.^(10)^

After the amputation, the rehabilitation program aims to regain autonomy for deambulation, with a prosthesis if possible, and also for daily activities, while taking care of the cognitive, emotional and social aspects.^(11,12)^

The rehabilitation of these patients is a challenge for the multidisciplinary team, because they suffer from other diseases associated with vascular disease, especially *diabetes mellitus*, and cardiovascular disorders, especially coronary heart disease, which can affect the survival of these individuals.^(8,9,13-15)^

Follow-up studies of this population show that reamputations are frequent,^(16-18)^ the abandonment rate of the use of the prosthesis is elevated,^(19)^ and the mortality rate is high.^(8,9, 20,21)^

The above data are worrisome and justify carrying out this study, in order to delineate the scenario of the department and to assist in the rehabilitation procedures.

## OBJECTIVE

To evaluate the prosthetization, during rehabilitation, the acceptance and abandonment rates of the prosthesis, after discharge, and their causes, as well as the mortality rate of lower limb amputee patients of vascular etiology.

## METHODS

This was a retrospective and cross-sectional study, based on the review of medical records of transtibial (TT) and transfemoral (TF) amputee patients of vascular etiology, followed at *Lar Escola São Francisco*, between 2003 and 2010. The review of the medical records was made from August to November 2011, and the interviews were conducted in December 2011. All patients or their relatives signed an Informed Consent, after the approval of the study by the Research Ethics Committee of the *Universidade Federal de São Paulo* (UNIFESP), protocol 0932/10.

The inclusion criteria for the study were: patients of both genders, regardless of age, lower limb amputees of vascular etiology followed at *Lar Escola* *São Francisco*, with TT and TF amputations, unilateral or bilateral, with or without associated diseases.

The exclusion criteria were: amputations due to other etiologies, non TF or TT lower limb amputations of vascular etiology, upper limb amputations, and patients with incomplete medical records.

The variables analyzed were: age, gender, side, level, unilateral or bilateral amputation, presence of associated diseases, prosthetization and, if so, acceptance or abandonment of the use of the prosthesis after discharge, and its reasons, and occurrences of deaths and their causes.

The method of data analysis made use of absolute and relative values, non-parametric statistical tests of equality of two proportions, 95% confidence interval and p value <0.05. We used the following software: Statistical Package for the Social Science (SPSS) V16, Minitab 15, and Excel Office 2007. For the analysis of logistics and odds ratio, the SAS System 9.0 software was used.

Between 2003 and 2010, a total of 425 patients underwent a preliminary medical evaluation at the Group of Amputations and Prostheses of the *Lar Escola* *São Francisco*. Among these, the distribution of etiologies was as follows: 45 (10.6%) trauma, 15 (3.5%) infection, 5 (1.2%) tumor, 5 (1.2%) congenital malformation, and 355 (83 5%) had vascular etiology. Of these, 45 had non TF and TT amputations (the levels selected for this study), and were excluded. The initial sample comprised 310 patients, of which 217 were prosthetized and 93 were not, due to clinical events or even for physical and functional disabilities. (Table 1)

**Table 1:** Data on review of medical records of amputee patients

Review 2003-2010	Total n (%)	Prosthetization	
		Yes	No
**n (%)**	**n (%)**
Number of patients	310 (100)	217 (70)	93 (30)
Male	205 (66.1)	147 (67.7)	58 (62.3)
Female	105 (33.9)	70 (32.3)	35 (37.7)
Mean age (in years)	61.81	62.19	61.98
Transfemoral	148 (47.8)	103 (47.4)	45 (48.4)
Unilateral right	64 (20.6)	47 (21.6)	17 (18.3)
Unilateral left	78 (25.3)	54 (24.9)	24 (25.8)
Bilateral	6 (1.9)	2 (0.9)	4 (4.3)
Transtibial	157 (50.6)	112 (51.5)	45 (48.4)
Unilateral right	70 (22.6)	51 (23.5)	19 (20.4)
Unilateral left	80 (25.8)	58 (26.7)	22 (23.6)
Bilateral	7 (2.2)	3 (1.3)	4 (4.3)
Bilateral assymetrical	5 (1.6)	2 (0.9)	3 (3.2)

For transfemoral amputee patients, the prescribed prostheses were modular, in steel or aluminum, with ischial support socket, suction valve, knee with brake and articulated foot; for transtibial amputee patients, the prosthesis were modular, in steel or aluminum, with KBM (condylar suspension) socket, and SACH (solid-ankle cushion heel) foot.

## RESULTS

A total of 195 patients (62.9% of the initial sample) were contacted and, of these, 151 had been prosthetized (77.4%), and 44 had not been prosthetized (22.6%) at the end of rehabilitation. Of the prosthetized, 54 were still using the protheses, 80 had abandoned them, and 17 had died. In the non-prosthetized group, 27 were using a wheelchair, and 17 had died (Table 2).

**Table 2:** Comparison of characteristics of prosthetized *versus* non-prosthetized

Variable	Prosthetization			p value
	No (n=44)	Yes (n=151)	Total (n=195)	
Mean age (SD)	65.2 (13.2)	61.0 (10.8)	61.9 (11.4)	0.029*
Gender				
Female	14 (31.8)	41 (27.2)	55 (28.2)	0.545
Male	30 (68.2)	110 (72.8)	140 (71.8)	
Transfemoral level	18 (40.9)	71 (47.0)	89 (45.6)	0.474
Unilateral right	7 (15.9)	33 (21.8)	40 (20.5)	
Unilateral left	9 (20.5)	37 (24.5)	46 (23.6)	
Bilateral	2 (4.5)	1 (0.7)	3 (1.5)	
Transtibial	26 (59.1)	80 (53.0)	106 (54.4)	
Unilateral right	11 (25.0)	38 (25.2)	49 (25.1)	
Unilateral left	13 (29.6)	40 (26.5)	53 (27.2)	
Bilateral	2 (4.5)	2 (1.3)	4 (2.1)	
Chronic obstructive pulmonary disease				>0.999#
No	44 (100.0)	150 (99.3)	194 (99.5)	
Yes	0 (0.0)	1 (0.7)	1 (0.5)	
Arterial hypertension				0.324
No	15 (34.1)	40 (26.5)	55 (28.2)	
Yes	29 (65.9)	111 (73.5)	140 (71.8)	
*Diabetes mellitus*				0.574
No	14 (31.8)	55 (36.4)	69 (35.4)	
Yes	30 (68.2)	96 (63.6)	126 (64.6)	
Coronary artery disease				0.243
No	34 (77.3)	128 (84.8)	162 (83.1)	
Yes	10 (22.7)	23 (15.2)	33 (16.9)	
Chronic renal failure				0.118#
No	40 (90.9)	146 (96.7)	186 (95.4)	
Yes	4 (9.1)	5 (3.3)	9 (4.6)	
Acute arterial obstruction				0.523#
No	40 (90.9)	141 (93.4)	181 (92.8)	
Yes	4 (9.1)	10 (6.6)	14 (7.2)	
Chronic arterial obstruction				0.429
No	28 (63.6)	86 (57.0)	114 (58.5)	
Yes	16 (36.4)	65 (43.0)	81 (41.5)	
Dyslipidemia				0.663
No	36 (81.8)	119 (78.8)	155 (79.5)	
Yes	8 (18.2)	32 (21.2)	40 (20.5)	
Congestive heart failure				0.735#
No	41 (93.2)	142 (94.0)	183 (93.8)	
Yes	3 (6.8)	9 (6.0)	12 (6.2)	
Smoking				0.108
No	35 (79.5)	101 (66.9)	136 (69.7)	
Yes	9 (20.5)	50 (33.1)	59 (30.3)	
Mean number of comorbidities (SD)	2.57 (1.04)	2.66 (1.07)	2.64 (1.06)	0.531**
Death				<0.001
No	27 (61.4)	134 (88.7)	161 (82.6)	
Yes	17 (38.6)	17 (11.3)	34 (17.4)	

Result of the χ^2^ test; * result of the Student *t* test; # result of the likelihood ratio; ** result of th Mann-Wihitney test. SD: standart deviation.

Of the 80 patients who abandoned the use of the prostheses, 56 were men and 24 women, mean age 62 years. Forty were unilateral TF amputees, 38 were unilateral TT amputees, 1 was a TF bilateral amputee, and 1 as a TT bilateral amputee. Seventy-one patients (88.75%) used walking aids (walker, crutches or cane) and only 9 (11.25%) reported that they walked without any assisting devices. The causes for abandoning the use of prostheses were: 13 (16.25%) patients had difficulty dressing, 16 (20%) were afraid of falling, 38 (47.5%) patients considered the prosthesis heavy, 5 (6,25%) were reamputated, 2 (2.5%) reported fatigue, 1 (1.25%) reported ghost-limb pain, 2 (2.5%) reported dizziness, 1 (1.25%) patient reported decompensation of blood pressure, 1 (1.25%) did not adapt to the prosthesis, and 1 (1.25%) had a cerebrovascular accident (stroke).

Of the 54 patients who continued using the prostheses, 43 were men, and the mean age was 61.8 years. Fifty-three were unilateral amputees (TT 32), and one was amputated bilaterally at the TF level. Thirty-six (66.7%) used walking aids, 12 (22.2%) did not use any resource besides the prosthesis, and 6 (11.1%) reported using a wheelchair for long distances.

Regarding the level of amputation and age, the results were similar among the groups as to contacted patients (89 TF, 106 TT, 61.9 years) and non-contacted patients (59 TF, 51 TT, 61.6 years). The frequency of males among contacted patients (n=140, 71.8%) was statistically higher than among non-contacted patients (n=65, 56.5%), with p=0.006.

The characteristics of the 195 contacted patients are shown in table 2, separating them into prosthetized and non-prosthetized individuals.

The mean age of the prosthetized patients was statistically lower than in the non-prosthetized group, with p=0.029 (Table 2).

In the prosthetized group, there were 17 deaths, of which 11 were men, and all had unilateral amputation (9 TF and 8 TT). The causes mentioned were: 13 patients with acute myocardial infarction (AMI), 2 patients after stroke, and 2 due to infection. In the non-prosthetized group, 17 deaths were reported, including 13 men. Of these deaths, 14 occurred in patients with unilateral amputation (9 TF, 5 TT) and 3 in patients with bilateral amputation (2 TF, TT 1), 12 with AMI, 4 after a stroke, one due to infection.

The mortality rate was statistically higher among prosthetized patients, with p <0.001, and the 34 deaths occurred, on average, 3.91 years after amputation (standard deviation – SD=1.79, median=3.61, minimum=0.66, and maximum=8.02) (Table 3).

**Table 3:** Death of prosthetized *versus* non-prosthetized patients

Prosthetization	Estimated mean time (years)	Standard error	95%CI		Deaths	Total	Death (%)	p value
			Inferior	Superior				
No	7.32	0.83	5.70	8.94	17	44	38.64	0.001
Yes	23.63	1.12	21.43	25.82	17	151	11.26	
Total	19.34	1.54	16.32	22.37	34	195	17.44	


95%CI: 95% interval confidence.

A logistic regression analysis was performed, as the model, considering as independent variables: age, gender, side and level of amputation, death and all the comorbidities separately. (Table 4)

**Table 4:** Logistic regression of prosthetization (dependent variable) *versus *age, gender, side and level of amputation, all comorbidities and death (independent variables)

Variable	GL	Estimated parameter	Standard error	Wald (χ^**2**^)	p value	Odds ratio	95%CI odds ratio (inferior)	95%CI odds ratio (superior)
Intercepto	1	10.19	757.80	0.00	0.99	-	-	-
Age	1	-0.06	0.02	9.22	0.002	0.94	0.90	0.98
Gender, female	1	-0.08	0.23	0.11	0.74	-	-	-
Side, right	1	0.94	0.40	5.71	0.02	10.79	1.50	77.49
Side, left	1	0.49	0.37	1.73	0.19	-	-	-
Level, transfemoral	1	0.08	0.21	0.14	0.71	-	-	-
COPD, absence	1	-6.46	757.80	0.00	0.99	-	-	-
AH, absence	1	-0.22	0.25	0.75	0.39	-	-	-
DM, absence	1	-0.05	0.28	0.04	0.85	-	-	-
Coronary artedy disease, absence	1	0.12	0.26	0.22	0.64	-	-	-
CRF, absence	1	0.82	0.41	3.99	0.05	5.14	1.03	25.59
AAO, absence	1	0.23	0.47	0.23	0.63	-	-	-
CAO, absence	1	-0.26	0.24	1.16	0.28	-	-	-
Dyslipidemia, absence	1	-0.19	0.26	0.53	0.47	-	-	-
CHF, absence	1	-0.52	0.44	1.39	0.24	-	-	-
Smoking, absence	1	-0.41	0.28	2.10	0.15	-	-	-
Survived	1	0.91	0.24	14.94	0.0001	6.16	2.45	15.50
Wald test								29.17
p value, global								0.02
AIC								203.05

Intercepto: model constant; FG: freedom grade; COPD: chronic obstructive pulmonar disease; AH: arterial hypertension; DM: *diabetes mellitus*; CRF: chronic renal failure; AAO: acute arterial occlusion; CAO: chronic arterial occlusion; CHF: congestive heart failure; AIC: Akaike information criteria; 95%CI: 95% interval confidence.

Statistically significant effects of age (p value=0.002), right side (p value=0.02), chronic renal failure (CRF) (p value=0.05), and death (p value=0.0001) were detected.

Age showed a negative parameter estimate (-0.06), indicating that the higher the age, the lower the probability of prosthetization. This could also be observed by the value of the odds ratio, which was <1 (0.94). In this case, each decrease of one year of age increased by 1.06-fold the probability of prosthetization.

There was a positive parameter estimate for the right side (0.94), indicating that the amputation of the right side increased the chance of the patient being prosthetized. The odds ratio obtained was 10.79, indicating that a right side amputation increased by about ten times this probability.

We observed a positive parameter estimate for the absence of CRF (0.82), indicating that not having CRF increased the chance of the patient being prosthetized. The odds ratio obtained was 5.14, indicating that not having CRF increased by about five times the chance of the patient being prosthetized.

In relation to death, there was a statistically significant parameter for survival (0.91), indicating that if the patient survives, it is more probable that this patient is a prosthetized individual. The odds ratio obtained was 6.16, indicating that a patient who survived was about six times more likely to be a prosthetic patient.

**Figure 1:**
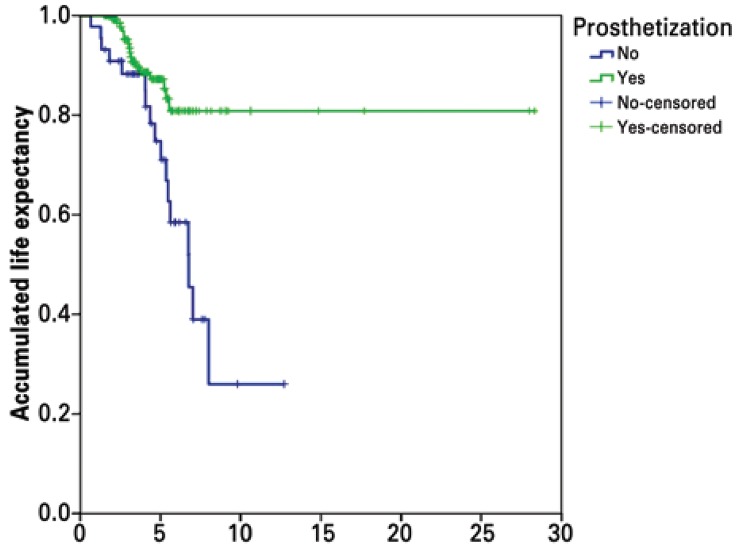
Kapla-Meier life expectancy graphic

The life expectancy of non-prosthetized patients was lower than that of the prosthetized ones, as shown in figure 1.

## DISCUSSION

Peripheral arterial diseases remain the main cause of lower limb amputation in the world, as described by several authors,^(2,3,13,18,22)^ and the same was observed in our service (83%), as this study demonstrated.

We observed a prevalence of males, representing 66% of the amputations, with a mean age of 62 years, similarly to the data from various published studies.^(1-6,11,15-28)^

As the results showed, the higher the age, the lower the chance of the patient being prosthetized. However, as observed in other studies,^(11,12,29-32)^ this isolated variable should not be an absolute contraindication for prosthetic prescription, but may influence the potential for success in walking.

There was a predominance of unilateral amputee patients, both TF (86 to 89;96.6%) and TT (102 in 106; 96.2%). Of the 195 contacted patients, 7 (3.5%) were bilateral amputee patients, with 3 prosthetized (1 TF and 2 TT) and 4 non-prosthetized (2 TF and TT 2). The reduced number of bilateral amputee patients certainly contributed to the results, both regarding the prosthetization and the number of deaths. The occurrence of bilateral amputation points to a greater severity of the peripheral arterial disease and a poorer prognosis for the indication and use of prostheses.^(33,34)^

The distribution of the amputation levels were similar between TT and TF, showing the occurrence of a greater number of amputations below the knee when compared to other studies conducted in the same service.^(23-27)^ This change can reveal some improvement in the treatment of the primary disease and in the understanding, by the team of surgeons, of the importance of preserving the knee joint for the rehabilitation of the patient, as noted in a recent publication.^(28)^

No articles were found associating the prosthetization of the lower limb with the amputation side, preventing a comparison with the results found in this study. In the present study, there was an association between the fact that the amputation occurred on the right side and the patient being prosthetized. The most plausible hypothesis is that the population of this study, in most cases, had right side dominance and, therefore, a better motor control in that side, but this information cannot be confirmed, as this piece of information was not collected or analyzed in the medical records or in the interviews with the patients.

The rehabilitation of these patients requires that the medical and therapeutic staff be very well trained to handle the clinical, physical, functional, emotional and social limitations resulting from the physical disability in the elderly population.^(29-30,33,34)^

The prosthetization at the end of the rehabilitation program was 70%, *i.e.*, below the average found in the literature, which ranges from 75 to 95%,^(11,35)^ but higher than that found in previous studies of the same service^(23-28)^ and also than that described by Pohjolainen et al.^(36)^

There was no statistically significant association between the comorbidities assessed and the prosthetization (p>0.05), except for chronic renal failure, which had a higher percentage of positivity in non-prosthetized patients. Not having CRF increased by about five times the chance of the patient being prosthetized. It is known that there is great difficulty in compliance and participation of the dialysis amputee in rehabilitation programs, due to transportation difficulties, the need for a caregiver, and the clinical oscillations resulting from the hemodialysis. In this study, 4 of the 80 patients (5%) who abandoned the use of the prostheses had CRF, and none of the 54 who continued using the prostheses had CRF.

It could be observed that the greater the number of comorbidities, the lower the life expectancy of the patient, especially in the case of *diabetes mellitus*. In this study, among the population whose death occurred, diabetes was present in 67% of cases. This finding was confirmed in the studies of Stewart et al.^(37)^ and McWhinnie et al.,^(38)^ in which the presence of *diabetes mellitus* was shown as a marker of increased morbidity among amputee patients, as well as a risk factor for shorter life expectancy after amputation, with survival rates around 27% at 5 years in diabetics and 40% among non-diabetics. De Luccia et al.^(19)^ found even more alarming figures, with a survival rate at 5 years of 45% in diabetics and of 85% among patients with peripheral vascular disease, with a six times higher risk of mortality in diabetics. A study conducted in Denmark showed that the risk of not surviving 30 days after amputation was six times higher in patients with four or five comorbidities, compared to those who had zero or one associated disease.^(9)^

The life expectancy (mean=3.9 years) was lower in non-prosthetized patients than in prosthetized patients, and this did not differ much from the findings of Nagashima et al.,^(1)^ Kulkarni et al.^(15) ^and Stewart et al.^(37)^

In this study, the rate of death was statistically lower in prosthetized patients (p<0.001).

The main cause of death in both groups was AMI (72%), confirming the literature.^(35-37,39)^ Colin and Collin^(35)^ observed a mortality rate of 45% within 2 years, and 75% in 4 years, 78% of cases due to AMI. Stewart et al.^(37)^ observed a mortality of 60%, with 73% resulting from AMI, and even higher in patients with *diabetes mellitus.* In another study by Stewart and Jain,^(39)^ heart disease accounted for 51% of causes of death among lower limb amputee patients. A study conducted in Rio de Janeiro^(13)^ with 50 amputee patients showed that 36 died in 6 years, of which 22 (61%) in the first year after the amputation, especially for heart problems.

It is interesting to note that only 13% of patients were known coronary artery disease carriers, differing from that found in other studies in which such values reached 30% at the time of the amputation.^(12,14,17,18)^

This finding points to the importance of a cardiac evaluation prior to the prescription of the prosthesis and justify the decision of not indicating its use in patients who have not been cleared by cardiologists after specific evaluations and exams, because it is known that there is a cardiac overload during the use of the prosthesis.^(30,35)^

After discharge from rehabilitation, an elevated abandonment rate of the prosthesis (62.5%) was observed, much higher than that found by Pohjolainen et al.,^(36)^ who found an abandonment rate of the prostheses of 8% during a follow-up of one year, but lower than that found by McWhinnie et al.,^(38) ^who showed an abandonment rate of 69% in 5 years, with a decrease in the daily use of the prosthesis from 85% to 31%.

It was observed that many patients reported being more independent with the use of a wheelchair than the prosthesis, especially due to its weight and the difficulty of putting it on, always needing the help of others, in addition to the fact that they felt safer and less fearful of falling.

The rate and the reasons for abandoning the prosthesis that we observed should assist the rehabilitation team in making decisions. One factor that must be considered and studied in the future is the need of the use of walking aids. In this study, the use of walking aids was more prevalent among patients who had abandoned the use of the prosthesis, among whom 70 of 80 used a cane or a walker or crutches, and this may be a marker of poor prognosis.

### Limitations of the study

There were no data on the causes of the indications for amputations (critical limb ischemia, infected ulcers, osteomyelitis, compartmental syndrome, etc.), for there was no access to the medical records of the patients during hospitalization. There was great difficulty in collecting data by telephone, due to the length and complexity of the research protocol, especially for answers regarding the use of the prosthesis.

The loss to follow-up was relevant, and we contacted 195 of 310 patients (62.9%).

Thus, an outpatient semiannual monitoring of the rehabilitated individuals should be taken in consideration in order to maintain the data from the follow-up after discharge from rehabilitation updated.

## CONCLUSION

Lower limb prosthetization of amputee patients of vascular etiology during rehabilitation was high, but the continued use of the prosthesis was low after completion of treatment. The mortality and early death rates of these patients were high, especially among diabetics.
