# Supplementary material for: Use of prostheses in lower limb amputee patients due to peripheral arterial disease
Source: Einstein (Sao Paulo). 2014 Oct-Dec;12(4):440–6. doi: 10.1590/S1679-45082014AO3132 (PMC4879909; doi:10.1590/S1679-45082014AO3132)
Supplement: Translation

# Translation: Uso de próteses em amputados de membros inferiores por doença arterial periférica

**Authors:** Therezinha Rosane Chamlian

**Affiliations:** 1 Escola Paulista de Medicina, Universidade Federal de São Paulo, São Paulo, SP, Brasil;; Lar Escola São Francisco, São Paulo, SP, Brasil.

**Keywords:** Extremidade inferior/cirurgia, Doença arterial periférica/complicações, Amputação/reabilitação, Amputados/reabilitação, Próteses e implantes, Mortalidade

## Abstract

**Objetivo:**

Avaliar a protetização, durante a reabilitação, e a manutenção do uso da prótese, e o índice de abandono da mesma após a alta, bem como a mortalidade dos pacientes amputados de membros inferiores por doença arterial periférica.

**Métodos:**

Estudo retrospectivo e transversal com pacientes amputados de membros inferiores nos níveis transtibial e transfemoral de etiologia vascular. A amostra foi composta por 310 pacientes (205 homens e 105 mulheres, média de idade de 61,8 anos), nos níveis transfemoral (142) e transtibial (150), unilateralmente ou bilateralmente (18). Foram protetizados 217 pacientes e 93 não. Foram utilizados testes estatísticos não paramétricos de igualdade de duas proporções, intervalo de confiança para média de 95% (IC95%) e valor de p<0,05

**Resultados:**

**. ** Dos 195 pacientes contatados, 151 haviam sido protetizados e 44 não. Dos protetizados, 54 mantinham-se usando suas próteses, 80 haviam abandonado o uso e 17 faleceram. No grupo dos não protetizados, 27 continuavam usando cadeira de rodas e 17 tinham evoluído para óbito. A mortalidade é estatisticamente maior nos pacientes não protetizados e os 34 óbitos ocorreram, em média, após 3,91 anos da amputação. O tempo de sobrevida dos pacientes não protetizados foi menor que o dos protetizados.

**Conclusão:**

A protetização de pacientes amputados de membros inferiores de etiologia vascular durante a reabilitação é alta, mas a manutenção do uso da prótese é baixa após o término do tratamento. A mortalidade desses pacientes é elevada e precoce, principalmente entre os diabéticos.

## INTRODUÇÃO

O número de pacientes amputados de membros inferiores por doença arterial periférica é elevado no Japão, Estados Unidos, Europa e Brasil, sendo a população mais acometida a idosa.^(1-6)^

Apesar dos avanços da Medicina e da ênfase na prevenção de doenças, as amputações continuam muito prevalentes no mundo e há previsão de que, em 2050, esse índice alcance 3,6 milhões de pessoas só nos Estados Unidos.^(7)^

As taxas de mortalidade 1 mês após a amputação são elevadas, variando de 15 a 30%.^(8,9)^ Após 1 ano, podem atingir índices superiores a 50%^(9)^ e, após 5 anos, podem chegar até 74%.^(10)^

Após a amputação, o programa de reabilitação visa recuperar a autonomia para locomoção, se possível com prótese, e para as atividades da vida diária, sem deixar de cuidar dos aspectos cognitivos, emocionais e sociais.^(11,12)^

A reabilitação desse grupo de pacientes é um desafio para a equipe multiprofissional, pois eles são portadores de outras doenças associadas à doença vascular, com destaque para o *diabetes mellitus*, e o comprometimento cardiológico, principalmente a doença coronariana, que podem interferir na sobrevida desses indivíduos.^(8,9,13-15)^

Estudos de seguimento dessa população mostram que as reamputações são frequentes,^(16-18)^ o abandono do uso da prótese é elevado^(19)^ e a taxa de mortalidade é alta.^(8,9^, ^20,21)^

Os dados referidos acima são preocupantes e justificam a realização deste estudo, a fim de retratar o cenário do serviço e de auxiliar nas condutas de reabilitação.

## OBJETIVO

Avaliar a protetização, durante a reabilitação, a manutenção do uso da prótese, e o índice de abandono da mesma, após a alta, e suas causas, bem como a mortalidade dos pacientes amputados de membros inferiores de etiologia vascular.

## MÉTODOS

Estudo retrospectivo e transversal, realizado com base na revisão dos prontuários de pacientes amputados de membros inferiores nos níveis transtibial (TT) e transfemoral (TF) de etiologia vascular, acompanhados no Lar Escola São Francisco entre 2003 e 2010. A revisão dos prontuários foi feita de agosto a novembro de 2011, e as entrevistas foram feitas em dezembro de 2011. Todos os pacientes ou familiares assinaram o Termo de Consentimento Livre e Esclarecido, após aprovação do estudo pelo Comitê de Ética em Pesquisa da Universidade Federal de São Paulo (UNIFESP), protocolo 0932/10.

Os critérios de inclusão no estudo foram: pacientes de ambos os sexos, independente da faixa etária, amputados de membros inferiores de etiologia vascular acompanhados no Lar Escola São Francisco, com amputação TT e TF, uni ou bilateralmente, com ou sem doenças associadas.

Os critérios de exclusão foram amputados de outras etiologias, de membros inferiores de etiologia vascular com níveis que não TF ou TT, de membros superiores e pacientes com prontuários incompletos.

As variáveis analisadas foram: idade, sexo, lado, nível e uni ou bilateralidade da amputação, presença de doenças associadas, protetização e, em caso afirmativo, manutenção do uso ou abandono da prótese após alta, e seus motivos e ocorrências de óbitos e suas causas.

O método de análise dos dados foi feito por meio de valores absolutos e relativos, e testes estatísticos não paramétricos de igualdade de duas proporções, intervalo de confiança para média de 95% e valor de p<0,05. Foram utilizados os *softwares*: *Statistical Package for the Social Science* (SPSS) V16, Minitab 15 e Excel Office 2007. Para as análises de regressão logística e *odds ratio*, foi utilizado o *software* *The SAS System 9.0.*

Entre 2003 e 2010, 425 pacientes passaram em consulta médica inicial no Grupo de Amputações e Próteses do Lar Escola São Francisco. Destes, a distribuição das etiologias era a seguinte: 45 (10,6%) trauma, 15 (3,5%) infecção, 5 (1,2%) tumor, 5 (1,2%) malformação congênita e 355 (83,5%) apresentavam etiologia vascular. Destes, 45 apresentavam níveis de amputação diferentes do TF e TT, selecionados para este estudo, e foram excluídos. A amostra inicial foi composta por 310 pacientes, sendo que 217 foram protetizados e 93 não o foram, por intercorrências clínicas ou mesmo por incapacidade física e funcional (Tabela1).

**Tabela 1:** Dados da revisão de prontuários dos pacientes amputados

Revisão 2003-2010	Total n (%)	Protetização	
		Sim	Não
		n (%)	n (%)
Número de pacientes	310 (100)	217 (70)	93 (30)
Masculino	205 (66,1)	147 (67,7)	58 (62,3)
Feminino	105 (33,9)	70 (32,3)	35 (37,7)
Média de idade (em anos)	61,81	62,19	61,98
Transfemoral	148 (47,8)	103 (47,4)	45 (48,4)
Unilateral direita	64 (20,6)	47 (21,6)	17 (18,3)
Unilateral esquerda	78 (25,3)	54 (24,9)	24 (25,8)
Bilateral	6 (1,9)	2 (0,9)	4 (4,3)
Transtibial	157 (50,6)	112 (51,5)	45 (48,4)
Unilateral direita	70 (22,6)	51 (23,5)	19 (20,4)
Unilateral esquerda	80 (25,8)	58 (26,7)	22 (23,6)
Bilateral	7 (2,2)	3 (1,3)	4 (4,3)
Bilateral assimétrico	5 (1,6)	2 (0,9)	3 (3,2)

Para os amputados transfemorais, as próteses prescritas foram modulares em aço ou alumínio, com encaixe de apoio isquiático, válvula de sucção, joelho com freio e pé articulado; para os amputados transtibiais, as próteses foram modulares em aço ou alumínio, com encaixe KBM (de suspensão condilar) e pé SACH (sigla do inglês *solid-ankle cushion heel*).

## RESULTADOS

Foram contatados 195 pacientes (62,9% da amostra inicial) e, destes, 151 tinham sido protetizados (77,4%) e 44 não foram protetizados (22,6%) ao término da reabilitação. Dos protetizados, 54 mantinham-se usando suas próteses, 80 tinham abandonado o uso, e 17 faleceram. No grupo dos não protetizados, 27 continuavam usando cadeira de rodas e 17 tinham evoluído para óbito (Tabela 2).

**Tabela 2:** Comparação das características dos pacientes protetizados *versus* não protetizados

Variável	Protetização			Valor de p
	Não (n=44)	Sim (n=151)	Total (n=195)	
Média da idade (DP)	65,2 (13,2)	61,0 (10,8)	61,9 (11,4)	0,029*
Gênero				
Feminino	14 (31,8)	41 (27,2)	55 (28,2)	0,545
Masculino	30 (68,2)	110 (72,8)	140 (71,8)	
Nível transfemoral	18 (40,9)	71 (47,0)	89 (45,6)	0,474
Unilateral direita	7 (15,9)	33 (21,8)	40 (20,5)	
Unilateral esquerda	9 (20,5)	37 (24,5)	46 (23,6)	
Bilateral	2 (4,5)	1 (0,7)	3 (1,5)	
Transtibial	26 (59,1)	80 (53,0)	106 (54,4)	
Unilateral direita	11 (25,0)	38 (25,2)	49 (25,1)	
Unilateral esquerda	13 (29,6)	40 (26,5)	53 (27,2)	
Bilateral	2 (4,5)	2 (1,3)	4 (2,1)	
Doença pulmonar obstrutiva crônica				>0,999#
Não	44 (100,0)	150 (99,3)	194 (99,5)	
Sim	0 (0,0)	1 (0,7)	1 (0,5)	
Hipertensão arterial sistêmica				0,324
Não	15 (34,1)	40 (26,5)	55 (28,2)	
Sim	29 (65,9)	111 (73,5)	140 (71,8)	
*Diabetes mellitus*				0,574
Não	14 (31,8)	55 (36,4)	69 (35,4)	
Sim	30 (68,2)	96 (63,6)	126 (64,6)	
Coronariopatia				0,243
Não	34 (77,3)	128 (84,8)	162 (83,1)	
Sim	10 (22,7)	23 (15,2)	33 (16,9)	
Insuficiência renal crônica				0,118#
Não	40 (90,9)	146 (96,7)	186 (95,4)	
Sim	4 (9,1)	5 (3,3)	9 (4,6)	
Obstrução arterial aguda				0,523#
Não	40 (90,9)	141 (93,4)	181 (92,8)	
Sim	4 (9,1)	10 (6,6)	14 (7,2)	
Obstrução arterial crônica				0,429
Não	28 (63,6)	86 (57,0)	114 (58,5)	
Sim	16 (36,4)	65 (43,0)	81 (41,5)	
Dislipidemia				0,663
Não	36 (81,8)	119 (78,8)	155 (79,5)	
Sim	8 (18,2)	32 (21,2)	40 (20,5)	
Insuficiência cardíaca congestiva				0,735#
Não	41 (93,2)	142 (94,0)	183 (93,8)	
Sim	3 (6,8)	9 (6,0)	12 (6,2)	
Tabagismo				0,108
Não	35 (79,5)	101 (66,9)	136 (69,7)	
Sim	9 (20,5)	50 (33,1)	59 (30,3)	
Número de comorbidades média (DP)	2,57 (1,04)	2,66 (1,07)	2,64 (1,06)	0,531**
Óbito				<0,001
Não	27 (61,4)	134 (88,7)	161 (82,6)	
Sim	17 (38,6)	17 (11,3)	34 (17,4)	

Resultado do teste χ2; *resultado do teste *t* Student; #resultado do teste da razão de verossimilhanças; **resultado do teste Mann-Whitney. DP: desvio padrão.

Dos 80 pacientes que abandonaram o uso das próteses, 56 eram homens e 24 mulheres, com média de idade de 62 anos. Quarenta eram amputados TF unilaterais, 38 TT unilaterais, 1 TF bilateral e 1 TT bilateral. Setenta e um pacientes (88,75%) utilizavam auxiliares de marcha (andador, muletas ou bengala) e apenas 9 (11,25%) referiram que deambulavam sem aditivos auxiliares. As causas para abandono do uso das próteses foram: 13 (16,25%) pacientes tinham dificuldade para vestir, 16 (20%) tinham medo de cair, 38 (47,5%) pacientes achavam a prótese pesada, 5 (6,25%) foram reamputados, 2 (2,5%) referiram cansaço, 1 (1,25%) referiu dor fantasma, 2 (2,5%) referiram tonturas, 1 (1,25%) paciente referiu descompensação da pressão arterial, 1 (1,25%) não se adaptou à prótese e 1 (1,25%) teve acidente vascular encefálico (AVE).

Dos 54 pacientes que mantiveram o uso da prótese, 43 eram homens, e a média da idade foi de 61,8 anos. Cinquenta e três eram amputados unilaterais (32 TT) e 1 era bilateralmente amputado no nível TF. Trinta e seis (66,7%) usavam auxiliares de marcha, 12 (22,2%) não utilizavam nenhum recurso além da prótese e 6 (11,1%) referiram utilizar cadeiras de rodas para longas distâncias.

Com relação ao nível de amputação e idade, houve semelhança entre os grupos de pacientes contatados (89 TF, 106 TT, 61,9 anos) e não contatados (59 TF, 51 TT, 61,6 anos). A frequência de contatados do sexo masculino (n=140, 71,8%) foi estatisticamente maior que nos não contatados (n=65, 56,5%), com p=0,006.

As características dos 195 pacientes contatados são apresentadas na tabela 2, separando-os em protetizados e não protetizados.

A média da idade dos pacientes protetizados foi estatisticamente menor que nos não protetizados, com p=0,029 (Tabela 2).

No grupo dos protetizados, foram constatados 17 óbitos, sendo 11 em homens e todos com amputação unilateral (9 TF e 8 TT). As causas citadas foram: 13 pacientes por infarto agudo do miocárdio (IAM), 2 pacientes após AVE e 2 por infecção. No grupo dos não protetizados, foram informados 17 óbitos, dos quais 13 em homens. Dentre esses óbitos, 14 ocorreram em pacientes com amputação unilateral (9 TF, 5 TT) e 3 em bilaterais (2 TF, 1 TT), sendo 12 por IAM, 4 após AVE, 1 por infecção.

A mortalidade foi estatisticamente maior nos pacientes não protetizados, com p<0,001, e os 34 óbitos ocorreram, em média, após 3,91 anos da amputação (desvio padrão − DP: 1,79, mediana 3,61, mínimo 0,66 e máximo 8,02) (Tabela 3).

**Tabela 3:** Ocorrência de óbitos entre pacientes protetizados e não protetizados

Protetização	Tempo médio estimado (anos)	Erro padrão	IC95%		Óbitos	Total	Óbitos (%)	Valor de p
			Inferior	Superior				
Não	7,32	0,83	5,70	8,94	17	44	38,64	0,001
Sim	23,63	1,12	21,43	25,82	17	151	11,26	

Total	19,34	1,54	16,32	22,37	34	195	17,44	

IC95%: intervalo de confiança de 95%.

Foi feita análise de regressão logística, conforme modelo que considerou como variáveis independentes idade, sexo, lado e nível da amputação, óbito e todas as comorbidades separadamente (Tabela 4).

**Tabela 4:** Regressão logística para protetização (variável dependente) *versus* idade, sexo, lado e nível da amputação, todas as comorbidades e óbito (variáveis independentes)

Variável	GL	Parâmetro estimado	Erro padrão	Wald (χ^**2**^)	Valor de p	*Odds ratio*	IC95% *odds ratio* (inferior)	IC95% *odds ratio* (superior)
Intercepto	1	10,19	757,80	0,00	0,99	-	-	-
Idade	1	-0,06	0,02	9,22	0,002	0,94	0,90	0,98
Sexo, feminino	1	-0,08	0,23	0,11	0,74	-	-	-
Lado, direito	1	0,94	0,40	5,71	0,02	10,79	1,50	77,49
Lado, esquerdo	1	0,49	0,37	1,73	0,19	-	-	-
Nível, transfemoral	1	0,08	0,21	0,14	0,71	-	-	-
DPOC, ausência	1	-6,46	757,80	0,00	0,99	-	-	-
HAS, ausência	1	-0,22	0,25	0,75	0,39	-	-	-
DM, ausência	1	-0,05	0,28	0,04	0,85	-	-	-
Coronariopatia, ausência	1	0,12	0,26	0,22	0,64	-	-	-
IRC, ausência	1	0,82	0,41	3,99	0,05	5,14	1,03	25,59
OAA, ausência	1	0,23	0,47	0,23	0,63	-	-	-
OAC, ausência	1	-0,26	0,24	1,16	0,28	-	-	-
Dislipidemia, ausência	1	-0,19	0,26	0,53	0,47	-	-	-
ICC, ausência	1	-0,52	0,44	1,39	0,24	-	-	-
Tabagismo, ausência	1	-0,41	0,28	2,10	0,15	-	-	-
Sobreviveu	1	0,91	0,24	14,94	0,0001	6,16	2,45	15,50
Wald teste								29,17
Valor de p, global								0,02
AIC								203,05

Intercepto: constante do modelo; GL: grau de liberdade; DPOC: doença pulmonar obstrutiva crônica; HAS: hipertensão arterial sistêmica; DM: *diabete mellitus; *IRC: insuficiência renal crônica; OAA: oclusão arterial aguda; OAC: oclusão arterial crônica; ICC: insuficiência cardíaca congestiva; AIC: critério de informação de Akaike; IC 95%: intervalo de confiança 95%.

Foram detectados efeitos estatisticamente significativos da idade (valor de p de 0,002), do lado direito (valor de p de 0,02), da insuficiência renal crônica (IRC) (valor de p de 0,05) e do óbito (valor de p de 0,0001).

A idade apresentou um parâmetro estimado negativo (-0,06), indicando que quanto maior a idade, menor a chance de ser protetizado. Isso também pode ser observado pelo valor de *odds ratio*, que foi <1(0,94). Nesse caso, cada ano de idade a menos incrementou em 1,06 vez a chance de ser protetizado.

Observou-se um parâmetro estimado positivo para o lado direito (0,94), indicando que a amputação ser do lado direito aumentou a chance do paciente ser protetizado. O *odds ratio* obtido foi de 10,79, indicando que esse fato aumentou em cerca de dez vezes essa chance.

Observou-se parâmetro estimado positivo para a ausência de IRC (0,82), indicando que não possuir IRC aumentou a chance de ser protetizado. O *odds ratio* obtido foi de 5,14, indicando que não possuir IRC aumentou em cerca de cinco vezes a chance de ser protetizado.

Em relação ao óbito, observou-se um parâmetro estatisticamente significativo para a sobrevivência (0,91), indicando que, se o paciente sobrevivesse, existiu mais chance de ter sido um paciente protetizado. O *odds ratio* obtido foi de 6,16, indicando que um paciente que sobreviveu tinha cerca de seis vezes mais chance de ser um paciente protetizado.

O tempo de sobrevida dos pacientes não protetizados foi menor que o dos protetizados, conforme demonstra a figura 1.

**Figura 1:** Gráfico de sobrevida de Kaplan-Meier

## DISCUSSÃO

As doenças arteriais periféricas permanecem como a principal etiologia de amputação de membros inferiores no mundo, conforme descrito por diversos autores^(2,3,13,18,22)^ e também o são em nosso serviço (83%), como demonstrou este estudo.

Observa-se prevalência do sexo masculino com 66% das amputações, com média de idade de 62 anos, dados semelhantes aos de diversos estudos já publicados.^(1-6,11,15-28)^

Conforme demonstraram os resultados, quanto maior a idade, menor a chance de ser protetizado. Entretanto, como observado em outros estudos,^(11,12,29-32)^ essa variável isolada não deve ser contraindicação absoluta para prescrição de prótese, mas vai influenciar no potencial de sucesso na marcha.

Houve predomínio das amputações unilaterais, tanto TF (86 em 89;96,6%) como TT (102 em 106; 96,2%). Dos 195 contatados, 7 (3,5%) eram bilateralmente amputados, sendo 3 protetizados (1 TF e 2 TT) e 4 não protetizados (2 TF e 2 TT). O numero reduzido de pacientes amputados bilateralmente certamente contribuiu com os resultados, tanto referentes à protetização, como quanto ao numero de óbitos. A ocorrência da bilateralidade da amputação já aponta para a maior severidade da doença arterial periférica e para o pior prognóstico para indicação e uso de próteses.^(33,34)^

A distribuição dos níveis de amputação foi semelhante entre TT e TF, demonstrando a ocorrência de maior número de amputações abaixo do joelho, quando comparados a outros estudos realizados no mesmo serviço.^(23-27)^ Essa mudança pode revelar alguma melhora na abordagem da doença primária e no entendimento, pela equipe de cirurgiões, da importância da preservação da articulação do joelho para a reabilitação do paciente, como já observado em recente publicação.^(28)^

Não foram encontrados artigos que associassem a protetização do membro inferior com o lado da amputação, prejudicando a comparação dos resultados encontrados aqui. Neste estudo, houve associação entre a amputação ter ocorrido do lado direito e o paciente ter sido protetizado. A hipótese mais plausível é a de que a população deste estudo, em sua maioria, teve dominância à direita e, por isso, melhor controle motor desse lado, mas essa informação não pode ser confirmada, pois tal dado não foi coletado dos prontuários analisados nem nas entrevistas realizadas com os pacientes.

A reabilitação desse grupo de pacientes exige que a equipe médica e terapêutica esteja muito bem treinada para lidar com as limitações clínicas, físicas, funcionais, emocionais e sociais, que se apresentam pela incapacidade física numa população idosa.^(29-30,33,34)^

A protetização ao término do programa de reabilitação foi de 70%, ou seja, esteve abaixo da média encontrada na literatura, que varia de 75 a 95%,^(11,35)^ mas superior ao encontrado em estudos anteriores do mesmo serviço^(23-28)^ e também ao descrito por Pohjolainen et al.^(36)^

Não houve associação estatisticamente significativa entre as comorbidades avaliadas e a protetização (p>0,05), com exceção de insuficiência renal crônica, que apresenta maior percentual de positividade nos pacientes não protetizados. Não possuir IRC aumenta em cerca de cinco vezes a chance de ser protetizado. Sabe-se que há muita dificuldade de adesão e participação do paciente amputado dialítico nos programas de reabilitação, tanto pelas dificuldades de transporte, necessidade de acompanhante e pelas oscilações clínicas consequentes à hemodiálise. Neste estudo, 4 dos 80 pacientes (5%) que abandonaram o uso da prótese tinham IRC e nenhum dos 54 pacientes que continuaram usando suas próteses tinha IRC.

Pôde-se observar que quanto maior o número de comorbidades, menor foi a sobrevida do paciente, especialmente tratando-se de *diabetes mellitus*. Neste estudo, dentre a população que evoluiu para óbito, o diabetes esteve presente em 67% dos casos. Tal dado se confirmou ao ser encontrado nos estudos de Stewart et al.^(37)^ e McWhinnie et al.,^(38)^ nos quais a presença de *dia* *betes mellitus* se mostrou como marcador de maior morbidade entre os pacientes amputados, bem como fator de risco de menor sobrevida após amputação, com taxa de sobrevida em torno de 27% em 5 anos nos diabéticos e de 40% entre os não diabéticos. De Luccia et al.^(19)^ encontraram valores ainda mais alarmantes, com taxa de sobrevida em 5 anos de 45% nos diabéticos e de 85% entre portadores de doença vascular periférica, com risco de mortalidade seis vezes maior nos diabéticos. Estudo realizado na Dinamarca mostrou que o risco de não sobreviver após 30 dias da amputação foi seis vezes maior no paciente com quatro ou cinco comorbidades, quando comparado ao que tivesse zero ou uma doença associada.^(9)^

O tempo de sobrevida dos pacientes (média de 3,9 anos) foi menor nos não protetizados do que nos protetizados e não diferiu muito dos achados de Nagashima et al.,^(1)^ Kulkarni et al.^(15)^e Stewart et al.^(37)^

Neste estudo, a frequência de óbito foi estatisticamente menor nos pacientes protetizados (p<0,001).

A principal causa de morte em ambos os grupos foi o IAM (72%), corroborando os dados da literatura.^(35-37,39)^ Colin e Collin^(35)^ observaram taxa de mortalidade de 45% em 2 anos e de 75% em 4 anos, sendo 78% dos casos decorrentes de IAM. Stewart et al.^(37)^ observaram mortalidade de 60% com 73% decorrente de IAM, sendo maior ainda nos pacientes com *diabetes mellitus*. Em outro estudo realizado por Stewart e Jain,^(39)^ as doenças do coração foram responsáveis por 51% das causas de morte entre amputados de membros inferiores. Estudo realizado no Rio de Janeiro^(13)^ com 50 amputados demonstrou que 36 faleceram em 6 anos, sendo 22 (61%) no primeiro ano de amputação, principalmente por problemas cardíacos.

Foi interessante constatar que apenas 13% dos pacientes eram sabidamente coronariopatas, diferindo do encontrado em outros estudos, nos quais tais valores chegaram a 30% no momento da amputação.^(12,14,17,18)^

Tal dado chama a atenção para a importância da avaliação cardiológica previamente à prescrição da prótese e justifica a conduta do serviço de não indicar a protetização em pacientes que não tenham sido liberados pelos cardiologistas, após avaliações e testes específicos, pois é sabido que há sobrecarga cardíaca durante o uso da prótese.^(30,35)^

Após a alta da reabilitação, foi observado elevado índice de abandono da prótese (62,5%), muito superior ao encontrado por Pohjolainen et al.,^(36)^ que constataram taxa de abandono da prótese de 8% durante acompanhamento de 1 ano, mas inferior ao encontrado por McWhinnie et al*.,* ^(38) ^que apresentaram taxa de abandono de 69% em 5 anos, com o uso diário da prótese passando de 85% para 31%.

Observou-se que muitos pacientes relataram ser mais independentes com o uso de cadeira de rodas do que com a prótese, especialmente devido ao peso e a dificuldade em colocá-la, sempre necessitando da ajuda de terceiros, além do que se sentem mais seguros e menos temerosos de quedas.

O índice e os motivos de abandono da prótese observados devem auxiliar na tomada de decisões da equipe de reabilitação. Um fator que deve ser levado em consideração e estudado no futuro é a necessidade do uso de auxiliares de marcha. Neste estudo, foi mais prevalente entre os pacientes que abandonaram o uso da prótese, sendo que 70 dos 80 usavam andador ou muletas ou bengala, podendo ser um marcador de mau prognóstico.

### Limitações do estudo

Não existiram dados das causas das indicações das amputações (isquemia crítica do membro, úlceras infectadas, osteomielite, síndrome compartimental etc.), pois não houve acesso aos prontuários dos pacientes na fase hospitalar. Houve grande dificuldade para coletar os dados por telefone, devido à extensão e à complexidade do protocolo de investigação, principalmente nas respostas referentes ao uso da prótese.

A perda durante o seguimento foi relevante, sendo possível o contato com 195 de 310 pacientes (62,9%).

Assim, deve-se considerar fazer acompanhamento ambulatorial semestral dos indivíduos reabilitados, a fim de manter atualizados os dados do seguimento após a alta da reabilitação.

## CONCLUSÃO

A protetização de pacientes amputados de membros inferiores de etiologia vascular durante a reabilitação foi alta, mas a manutenção do uso da prótese foi baixa após o término do tratamento. A mortalidade destes pacientes foi elevada e precoce, principalmente entre os diabéticos.
